# Comparative Valorization of Amazonian Fruit Wastes Reveals Açaí Seeds as a Promising Source of Antibacterial and Antioxidant Compounds

**DOI:** 10.1111/1750-3841.71477

**Published:** 2026-09-25

**Authors:** Lucas Figueiredo da Silva, Yasmin Lobato Salgado, Yanka Braga Palheta, Marta Chagas Monteiro, Ingryd Rodrigues Martins, Johnatt Allan Rocha de Oliveira

**Affiliations:** ^1^ Programa de Pós‐Graduação em Ciências Farmacêuticas (PPGCF) Instituto de Ciências da Saúde (ICS) Universidade Federal do Pará (UFPA) Belém Pará Brazil; ^2^ Faculdade de Farmácia (FACFAR), Instituto de Ciências da Saúde (ICS) Universidade Federal do Pará (UFPA) Belém Pará Brazil; ^3^ Laboratório de Higiene de Alimentos e Bioprocessos (LaHiBio), Faculdade de Nutrição (FANUT), Instituto de Ciências da Saúde (ICS) Universidade Federal do Pará (UFPA) Belém Pará Brazil; ^4^ Programa de Pós‐Graduação em Desenvolvimento Rural e Gestão de Empreendimentos Agroalimentares (PPDRGEA) Instituto Federal do Pará (IFPA) Castanhal Pará Brazil

**Keywords:** biomass, flavonoids, phenolic compounds, principal component analysis, waste valorization

## Abstract

Evaluation of the biological activity of food wastes is essential to identify promising bioactive compound sources for food applications. This study comparatively analyzed bioactive compounds and biological activities of Amazonian agro‐industrial fruit wastes: acerola pomace (AP), açaí seed (AS), mango peel (MP), and pupunha peel (PP), aiming at their valorization as natural antioxidants and antimicrobials. Extracts were obtained by dynamic maceration using ethanol and methanol, and thermal treatment using water and diluted sulfuric acid at mass/solvent ratios of 1:10 and 1:20 (w/v). Total phenolic content (TPC), total flavonoid content (TFC), antioxidant activity (AA), and major phenolic compound profile by HPLC‐DAD were determined. Antibacterial activity was assessed by broth microdilution. Extracts showing antimicrobial potential were further evaluated for cytogenetic safety using the *Allium cepa* assay. Ethanolic and methanolic AS extracts (ASE and ASM) at 1:10 (w/v) showed the highest TPC (2,252.20 and 2240.00 mg GAE/100 g, respectively). ASM showed the highest TFC (1112.31 mg/100 g), while ASE showed the highest AA (25661.90 µmol TE/100 g). Catechin was the predominant phenolic compound in ASE 1:10 (29.86 ± 0.64 mg/100 g). Only ASE and ASM showed antibacterial activity, with bactericidal effects against *Staphylococcus aureus* and bacteriostatic activity against *Proteus mirabilis* and *Pseudomonas aeruginosa*. ASM exhibited genotoxic and mitodepressive effects, whereas ASE demonstrated the lowest cytogenetic alterations at the bactericidal concentration. ASE represents a promising renewable source of bioactive compounds for high‐value products, supporting sustainable waste management.

**Practical Applications:**

Amazonian fruit residue extracts, especially those obtained from açaí seeds, showed potential as sustainable sources of natural antioxidant and antibacterial compounds. The ethanolic açaí seed extract may be explored as a functional ingredient or natural additive in food products, contributing to cleaner‐label formulations, improved oxidative stability, and extended shelf life. This study also highlights the importance of selecting extraction methods that combine bioactivity with biological safety for future food applications.

## 1 Introduction

Tropical fruit trees from the Amazon region represent a significant share of Brazil's biodiversity, positioning the country as a major producer of fruits of industrial relevance (Diaconeasa et al. 2023). In this context, fruit residues constitute a substantial fraction of agro‐industrial waste, consisting of organic materials generated at different stages of the food supply chain (Kumar et al. 2020). These residues are mainly composed of peels, seeds, and pomace, which are produced in large volumes, particularly by juice and pulp industries (Kandemir et al. 2022). The generation of such wastes is expected to intensify as global food production increases to meet the demands of a growing population (Fierascu et al. 2020). Consequently, the valorization of fruit residues has gained increasing attention as a sustainable strategy to reduce food waste (Aqilah et al. 2023).

Driven by the increasing demand for natural bioactive compounds, fruit by‐products have gained attention as valuable raw materials for food and pharmaceutical applications, making their valorization through bioactive compound recovery a promising strategy for the generation of value‐added bioproducts (Aqilah et al. 2023). These biomasses are rich in secondary plant metabolites, including phenolic compounds and, in particular, flavonoids, associated with antioxidant, antimicrobial, and antiproliferative effects (Haminiuk et al. 2012). Moreover, their generally low cytotoxicity makes them attractive candidates for pharmacological applications and for use as natural additives in food preservation, interest reinforced by the increasing resistance of foodborne microorganisms to synthetic preservatives (Jimenez‐Lopez et al. 2020).

Fruit wastes are predominantly lignocellulosic materials composed of cellulose, hemicellulose, and lignin, which confer structural rigidity and resistance to degradation. As a result, efficient recovery of bioactive compounds often requires the application of alternative extraction or pretreatment strategies (Elumalai et al. 2018). In addition to conventional alcoholic extractions, hydrothermal and dilute acid treatments conducted under elevated temperature and pressure conditions have been employed to promote the depolymerization of lignin and polysaccharides (Fernandes et al. 2017). These processes can generate extracts enriched with monosaccharides, phenolic compounds, furfural, and hydroxymethylfurfural, expanding the potential applications of the recovered fractions (de Oliveira et al. 2021).

Recent evidence has highlighted Amazonian fruit residues as valuable sources of bioactive compounds, reinforcing their potential for food, pharmaceutical, and biotechnological applications (Cruz et al. 2024). Despite the increasing scientific interest in these by‐products, markedly different extraction approaches have been employed, ranging from conventional solvent extraction to thermochemical processes, as well as distinct analytical methods and biological assays (Alves Borges et al. 2021; Conrado et al. 2025; Kučuk et al. 2024; Gomes de Souza et al. 2026), thereby limiting direct comparisons across the literature. Therefore, studies integrating different Amazonian fruit residues and diverse extraction strategies under the same experimental framework are essential to enable comparisons of their bioactive potential and identify the most promising biomasses for the development of value‐added products.

To address this gap, socio‐economically relevant fruit wastes from the Amazon region were investigated, including acerola pomace (AP; *Malpighia emarginata* DC), açaí seeds (AS; *Euterpe oleracea* Mart.), mango peels (MP; *Mangifera indica* L. ‘Espada’), and pupunha peels (PP; *Bactris gasipaes* Kunth.). These materials were used to produce ethanolic, methanolic, dilute acid, and aqueous extracts to comparatively evaluate the recovery of bioactive compounds using different extraction approaches applied to fruit waste biomass.

The objectives of this work were to quantify the total phenolic content (TPC) using the Folin–Ciocalteu method, determine the total flavonoid content (TFC) by aluminum complexation, assess the in vitro antioxidant activity (AA) through the DPPH radical scavenging assay, and analyze the major phenolic compound profile by HPLC‐DAD. In addition, the antimicrobial activity of the extracts was evaluated using the broth microdilution method. Extracts exhibiting relevant antimicrobial effects were further selected for the *Allium cepa* assay as a preliminary screening tool to assess cytogenetic safety and investigate potential cytotoxic and genotoxic effects.

## 2 Material and Methods

### 2.1 Chemicals and Reagents

2,2‐Diphenyl‐1‐picrylhydrazyl (DPPH), 6‐hydroxy‐2,5,7,8‐tetramethylchroman‐2‐carboxylic acid (Trolox), Folin–Ciocalteu reagent, gallic acid, and quercetin were obtained from Sigma–Aldrich (St. Louis, MO, USA). Acetic acid, aluminum chloride (AlCl_3_), ethanol (EtOH), methanol (MeOH), dimethyl sulfoxide (DMSO), hydrochloric acid (HCl), sodium carbonate (Na_2_CO_3_), and sulfuric acid (H_2_SO_4_) were purchased from Dinâmica Ltda (Indaiatuba, SP, Brazil). Chloramphenicol, Mueller–Hinton agar (MHA), and Mueller–Hinton broth (MHB) were acquired from Kasvi (São Paulo, SP, Brazil). Orcein and resazurin were obtained from INLAB (Vila Campestre, SP, Brazil).

### 2.2 Sample Preparation

Fresh samples of AP, AS, MP, and PP were collected from a local market in Belém, Pará, Brazil (1°27′08.3″ S, 48°30′13.2″ W), after pulp removal for commercialization. The material was dried in a forced‐air circulation oven (model 3 MedClave, São Paulo, SP, Brazil) at 50°C for 48 h. Subsequently, the dried material was ground in a knife mill (Star FT 50 Fortinox, Piracicaba, SP, Brazil), sieved through a 9‐mesh (2.0 mm) screen, and stored at −4°C until the extraction.

### 2.3 Extraction Procedure

Extractions were carried out using mass/solvent ratios of 1:10 and 1:20 (w/v), selected based on a previous study by de Oliveira et al. (2021). Alcoholic extracts were obtained by dynamic maceration using ethanol (70%; v/v) and pure methanol. The solvent was added to the samples in an Erlenmeyer flask, which were placed in a shaker incubator (AL‐222, AmericanLab) at 200 rpm and 60°C for 60 min (de Oliveira et al. 2022).

Dilute‐acid hydrothermal treatment was included as a comparative thermochemical strategy based on previous work demonstrating that hydrothermal pretreatment of açaí (*E. oleracea* Mart.) seeds can enhance enzymatic hydrolysis and sugar recovery (Oliveira et al. 2014; Oladzad et al. 2024). Under acidic conditions and elevated temperature, hydrolysis of acid‐labile bonds and partial disruption of lignin–carbohydrate associations may increase the accessibility of compounds associated with the plant cell‐wall matrix (Yuan et al. 2021). In the present study, the H_2_SO_4_ treatment was used primarily as a comparative extraction condition rather than as a directly food‐ready process.

**FIGURE 1:**
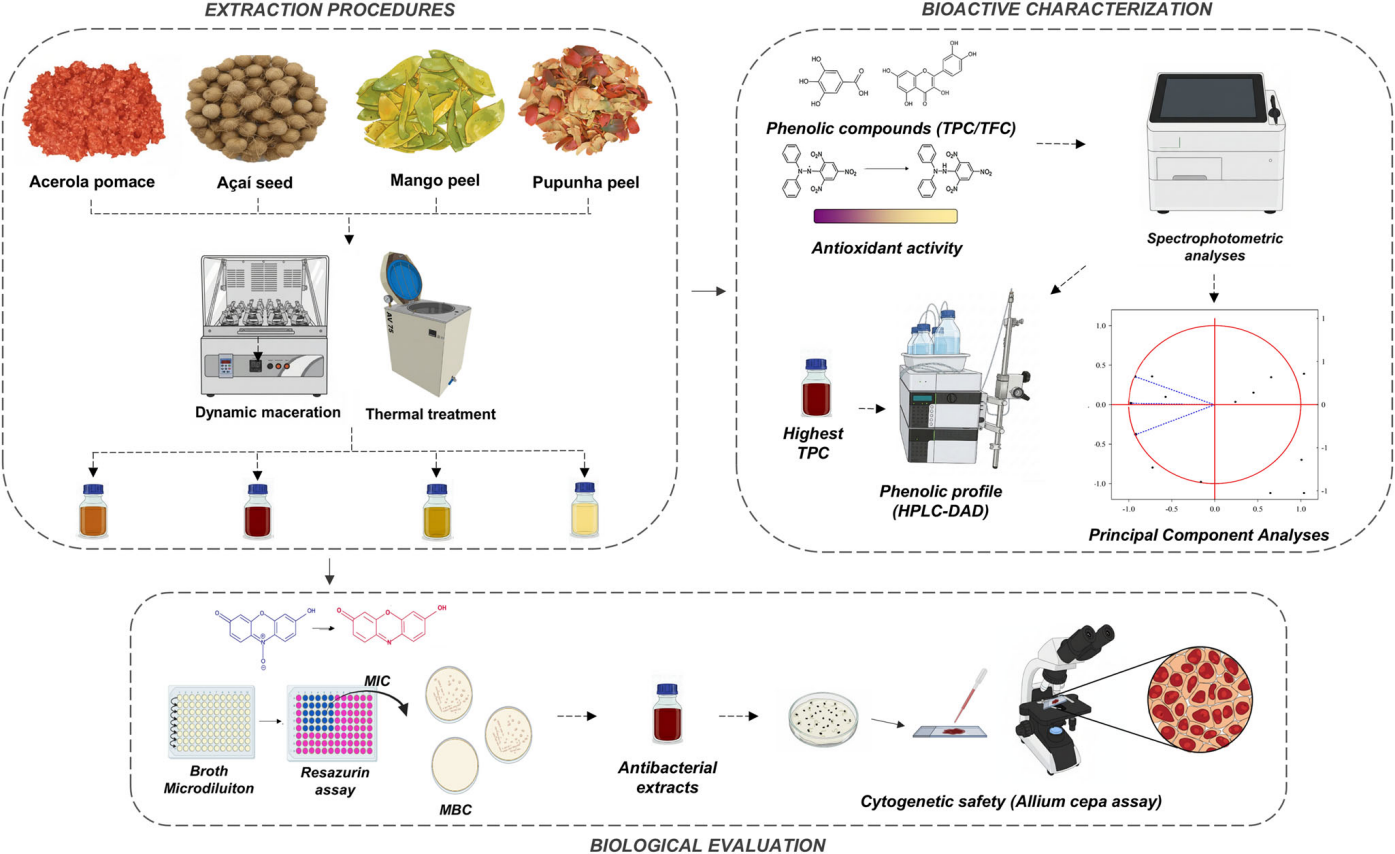
Experimental workflow for the extraction and biological evaluation of Amazonian agro‐industrial residue extracts.

Thus, dilute acid and aqueous extracts were produced by treatment with H_2_SO_4_ (1%; v/v) and deionized water, respectively. The solvent was added to the samples in glass tubes and subjected to a 60 min cycle at 121°C in a vertical autoclave (AISI 304, SOLAB, Piracicaba, SP, Brazil) (Oliveira et al. 2014). The resulting liquid extracts were filtered and concentrated using a rotary evaporator (SL‐126, SOLAB, Piracicaba, SP, Brazil) at 39°C. The dried extracts were stored at 4°C until further analyses (Figure 1).

### 2.4 Total Phenolic Content (TPC)

TPC was determined using the Folin–Ciocalteu method, carried out according to Singleton et al. (1999). For the analysis, 25 µL of the diluted extract was mixed with 125 µL of Folin–Ciocalteu 2 N (10%; w/v) and 100 µL of Na_2_CO_3_ (7.5%; w/v). The mixture was incubated for 2 h in the dark, and the sample absorbance was measured at 765 nm using an ELISA microplate reader (BK‐EL10C, OLABO, Shandong, China). A calibration curve was constructed in gallic acid equivalents (GAE) at concentrations of 10 to 300 µg/mL (*y* = 0.0067*x* + 0.0383; *R* ^2^ = 0.9963; LOD = 0.94 µg/mL; LOQ = 3.14 µg/mL). The results were expressed as mg GAE/100 g of dry extract.

### 2.5 Total Flavonoid Content (TFC)

TFC quantification was performed according to Pękal and Pyrzynska (2014). For the analysis, 115 µL of diluted extract was mixed with 115 µL of AlCl_3_ (2%; w/v). The mixture was incubated for 10 min in the dark, and the absorbance was measured at 425 nm using an ELISA microplate reader (BK‐EL10C, OLABO, Shandong, China). A calibration curve was constructed in quercetin equivalents (QE) at concentrations of 10 to 200 µg/mL (*y* = 0.0157*x* + 0.0031; *R* ^2^ = 0.9997; LOD = 0.24 µg/mL; LOQ = 0.81 µg/mL). The results were expressed as mg QE/100 g of dry extract.

### 2.6 Antioxidant Activity (AA)

To quantify the AA, the DPPH method was performed as described by Macedo et al. (2011). For the analysis, 50 µL of the diluted extract was mixed with 150 µL of the DPPH (0.2 mM). The mixture was incubated for 40 min in the dark, and the sample absorbance was measured at 520 nm using an ELISA microplate reader (BK‐EL10C, OLABO, Shandong, China). A calibration curve was constructed in Trolox equivalents (TE) at concentrations of 15 to 300 µmol/mL (*y* = ‐0.0021*x* + 0.5745; *R* ^2^ = 0.9903; LOD = 4.89 µg/mL; LOQ = 16.29 µg/mL). The data were expressed as µmol TE/100 g of dry extract.

### 2.7 Profile of Major Phenolic Compounds From ASE by HPLC‐DAD

The phenolic compound profile of ASE was determined by high‐performance liquidchromatography (HPLC). For preparation, the sample was resuspended using 10 mg in 10 mL of a mixture containing 95% of phase A and 5% of phase B, diluted (1:10; v/v), homogenized in a vortex mixer (VX‐38, IONLAB, Brazil) for 60 s, and filtered through a hydrophilic PTFE membrane with a pore size of 0.22 µm and a diameter of 13 mm (BioNaky, China).

The analyses were conducted using a Shimadzu Prominence HPLC system (Shimadzu Corp., Kyoto, Japan), equipped with a degassing unit (DGU‐20A5R), a pump (LC‐20AT), and a Rheodyne manual injector with a diode array detector (SPD‐M20A). Detection was monitored at 280 nm, with the spectrum configured to scan between 190 and 360 nm, following a modified method by Abe et al. (2007). Chromatographic separation was performed on an Agilent Polaris 3 C18‐A reversed‐phase analytical column (150 × 4.6 mm; particle size 3.0 µm; Agilent Technologies, CA, USA).

The injection volume was 20 µL, with a flow rate of 0.5 mL/min, a temperature of 40°C, and a total run time of 35 min. The mobile phase consisted of (phase A) ultrapure water (Millipore Milli‐Q, Millipore SAS) acidified with 0.1% trifluoroacetic acid (TFA, 99%, Sigma‐Aldrich, St. Louis, MO, USA) and (mobile phase B) acetonitrile (99.9%, Sigma‐Aldrich, St. Louis, MO, USA), using a linear gradient elution system with the following proportions: 17% B for 7 min, increasing to 25% B for 8 min, 35% B for 5 min, and 50% B for 3 min. For maintenance, the percentage of B was increased to 90% for an additional 9 min, and the initial conditions (B: 17%) were restored for 5 min.

The identification and quantification of catechin were conducted by comparing the retention time and UV–vis absorption spectrum of the catechin standard (99.9%, Sigma‐Aldrich, St. Louis, MO, USA). The chromatogram results were obtained using LabSolutions Lite software (Shimadzu UK Ltd., version 5.93). A calibration curve was constructed in catechin equivalent (CE) at concentrations ranging from 20 to 60 µg/mL (*y* = 495.366,66 *x* − 3.959.354,07; *R* ^2^ = 0.9975; LOD = 3.74 µg/mL; LOQ = 11.33 µg/mL). The results were expressed as mg CE/100 g of dry extract.

### 2.8 In Vitro Antibacterial Activity Assessment

#### 2.8.1 Bacterial Strains

American Type Culture Collection (ATCC) bacterial strains of *Escherichia coli* (ATCC 25922), *Salmonella typhimurium* (ATCC 14028), *Staphylococcus aureus* (ATCC 25923), *Proteus mirabilis* (ATCC 29906), and *Pseudomonas aeruginosa* (ATCC 27853) were used in this study.

#### 2.8.2 Minimum Inhibitory Concentration (MIC) and Minimum Bactericidal Concentration (MBC) Determination

MICs were determined using broth microdilution, according to CLSI document M07‐A11 (CLSI 2018). The extracts were diluted in DMSO (10%; v/v), and 100 µL was transferred to microplates with MHB. Serial twofold dilutions were performed, obtaining solutions with concentrations of 8–0.06 mg/mL. For the bacterial suspension, three to five isolated colonies were taken from a 24‐h bacterial culture and inoculated into MHB. The suspensions were standardized spectrophotometrically at 625 nm to match a 0.5 McFarland standard (1.5 × 10^8^ CFU/mL). Subsequently, 100 µL of the standardized inoculum was added to each well to achieve a final concentration of 5 × 10^5^ CFU/mL. Chloramphenicol (1 mg/mL) was used as the positive control.

Microplates were incubated at 36.5°C for 24 h in a bacteriological oven (TLK 48 DeLeo, Porto Alegre, RS, Brazil). After incubation, 30 µL of resazurin solution (0.01%, w/v) was added, followed by an additional incubation of 3 h. MIC values were defined as the lowest extract concentrations showing no color change. Minimum bactericidal concentrations (MBC) were determined according to Andrews (2001) by plating 10 µL aliquots from inhibitory wells onto MHA plates, followed by incubation at 36.5°C for 24 h. MBC was defined as the lowest concentration showing no visible bacterial growth.

### 2.9 *Allium cepa* Assay

Cytogenetic safety was evaluated using a bioactivity‐guided approach. Following antimicrobial screening, only extracts exhibiting bactericidal activity within the tested concentration range were selected for further analysis. The *Allium cepa* assay was performed according to Fiskesjö et al. (1985).

Ten onion seeds were placed in Petri dishes lined with filter paper moistened with deionized water until root lengths reached approximately 1 cm. Germinated seeds were exposed for 24 h in the dark and at room temperature to the selected extracts diluted in DMSO (1%; v/v) at a final concentration of 2 mg/mL, corresponding to the lowest concentration associated with bactericidal activity. DMSO (1%, v/v) was used as the negative control, while colchicine served as the positive control due to its well‐established genotoxic activity (Barman et al. 2020), applied at the same concentration as the test samples.

After exposure, roots were fixed in ethanol–acetic acid (3:1; v/v; Carnoy's fixative) for 24 h, washed with distilled water, and hydrolyzed with HCl (1 N) for 5 min. Samples were stained with orcein (2%; w/v) for 10 min. Approximately 2 mm of the apical meristem was excised, squashed on microscope slides, and covered with coverslips for cytological analysis. For each treatment, three slides were prepared, and 2000 cells were analyzed per slide, totaling 6000 cells per treatment. The mitotic index (MI) was calculated to assess cytotoxicity, and the frequency of chromosomal aberrations (CA) was determined to evaluate genotoxic effects, along with the identification of major cellular alterations.

### 2.10 Statistical Analysis

Principal component analysis (PCA) was performed using the correlation matrix of the mean TPC, TFC, and AA values to evaluate the relationships among these variables and the extract samples (AP, AS, MP, and PP). The first two principal components were used to construct the PCA biplot. All experiments were performed in triplicate, and results were expressed as mean ± standard deviation. Data were analyzed by one‐way analysis of variance (ANOVA), followed by Tukey's post hoc test (*p* ≤ 0.05), using Minitab 21 software (State College, PA, USA).

## 3 Results and Discussion

### 3.1 Bioactive Compounds and Antioxidant Activity of Extracts

**FIGURE 2:**
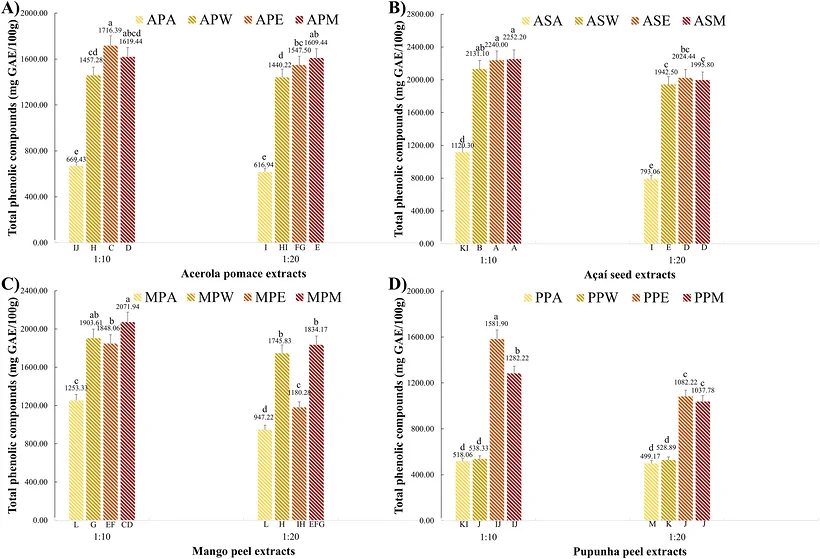
Total phenolic content (TPC) of extracts obtained from Amazonian agro‐industrial residues: acerola pomace (A), açai seed (B), mango peel (C), and pupunha peel (D). Experiments were presented as mean ± standard deviation. Different lowercase letters (a, b) indicate significant differences (*p* < 0.05) among the means of extracts obtained from the same residue (AP, AS, MP, and PP) by Tukey's test. Different uppercase letters (A, B) indicate significant differences (*p* < 0.05) among the means of the evaluated extracts by Tukey's test. APA, acerola pomace acid extract; APE, acerola pomace ethanolic extract; APM, acerola pomace methanolic extract; APW, acerola pomace water extract; ASA, açaí seed acid extract; ASE, açaí seed ethanolic extract; ASM, açaí seed methanolic extract; ASW, açaí seed water extract; GAE, gallic acid equivalent; MPA, mango peel acid extract; MPE, mango peel ethanolic extract; MPM, mango peel methanolic extract; MPW, mango peel water extract; PPA, pupunha peel acid extract; PPE, pupunha peel ethanolic extract; PPM, pupunha peel methanolic extract; PPW, pupunha peel water extract; TPC, total phenolic content.

**FIGURE 3:**
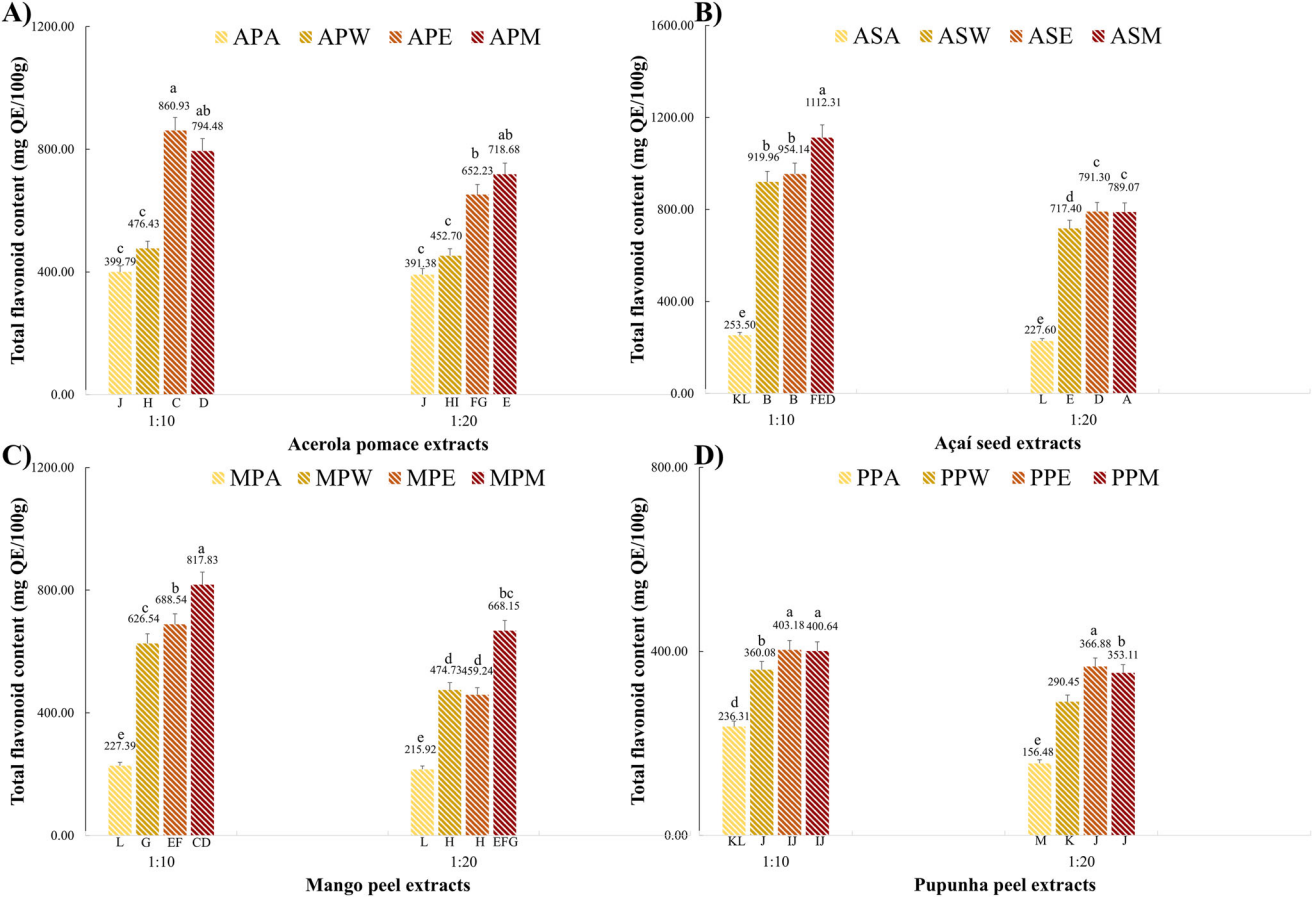
Total flavonoid content (TFC) of extracts obtained from Amazonian agro‐industrial residues: acerola pomace (A), açai seed (B), mango peel (C), and pupunha peel (D). Experiments were presented as mean ± standard deviation. Different lowercase letters (a, b) indicate significant differences (*p* < 0.05) among the means of extracts obtained from the same residue (AP, AS, MP, and PP) by Tukey's test. Different uppercase letters (A, B) indicate significant differences (*p* < 0.05) among the means of the evaluated extracts by Tukey's test. APA, acerola pomace acid extract; APE, acerola pomace ethanolic extract; APM, acerola pomace methanolic extract; APW, acerola pomace water extract; ASA, açaí seed acid extract; ASE, açaí seed ethanolic extract; ASM, açaí seed methanolic extract; ASW, açaí seed water extract; MPA, mango peel acid extract; MPE, mango peel ethanolic extract; MPM, mango peel methanolic extract; MPW, mango peel water extract; PPA, pupunha peel acid extract; PPE, pupunha peel ethanolic extract; PPM, pupunha peel methanolic extract; PPW, pupunha peel water extract; QE, quercetin equivalent; TFC, total flavonoid content.

As shown in Figures 2 and 3, TPC and TFC were significantly influenced by both biomass type and extraction conditions (*p* < 0.05). Across all treatments, AS extracts exhibited the highest bioactive concentrations, followed by MP, AP, and PP in decreasing order (*p* < 0.05).

Conventional ethanol and methanol extractions performed at a mass/solvent ratio of 1:10 (w/v) maximized phenolic and flavonoid recovery (*p* < 0.05), whereas increasing the solvent volume to 1:20 (w/v) resulted in significantly lower concentrations (*p* < 0.05), indicating a dilution effect rather than enhanced mass transfer. In contrast, acid and hydrothermal treatments significantly reduced TPC and TFC (*p* < 0.05), suggesting possible phenolic degradation or structural modification under more severe extraction conditions.

Under hydrothermal acidic conditions, acid‐labile glycosidic bonds in phenolic conjugates may be hydrolyzed, releasing the corresponding aglycones (Nuutila et al. 2002). da Silva et al. (2025) also reported that some flavonoids, with resistant chemical structures, may remain unchanged or even increase in environments containing acids. Partial disruption of lignin–carbohydrate associations may also increase the accessibility of matrix‐associated compounds (Yuan et al. 2021). Therefore, the expected soluble products may include phenolic acids released from the plant matrix and flavonoid aglycones formed through the hydrolysis of phenolic glycosides. However, excessive treatment severity may induce degradation or structural transformation of thermolabile phenolic compounds and generate secondary degradation products (Zalidis et al. 2025). Because the acid‐treated extracts were not subjected to targeted chromatographic characterization, the formation of these expected products was not experimentally confirmed.

For AP (Figure 2A), APE 1:10 achieved the highest TPC (1,716.39 mg GAE/100 g), statistically similar to APM 1:10 (*p* > 0.05), whereas APA 1:20 exhibited the lowest value (616.94 mg GAE/100 g) (*p* < 0.05). For AS (Figure 2B), ASM 1:10 (2,252.20 mg GAE/100 g) and ASE 1:10 (2,240 mg GAE/100 g) represented the most effective extraction conditions, with no difference between them (*p* > 0.05). The ASA 1:20 had the lowest TPC (793.06 mg GAE/100 g), differing from the other AS extracts (*p* < 0.05).

Among MP extracts (Figure 2C), TPC ranged from 947.22 to 2,071.94 mg GAE/100 g (*p* < 0.05). The highest TPC was observed for MPM 1:10 (2,071.94 mg GAE/100 g), which did not differ from MPW 1:10 (*p* > 0.05). In contrast, MPA 1:20 exhibited the lowest value (947.22 mg GAE/100 g) (*p* < 0.05). Unlike the pattern observed for AP, hydrothermal treatment promoted phenolic recovery comparable to methanolic extraction, suggesting a distinct behavior for MP.

For PP (Figure 2D), the highest TPC was observed for PPE 1:10 (1,581.9 mg GAE/100 g), whereas PPA 1:20 presented the lowest concentration (499.17 mg GAE/100 g), differing significantly from the other PP extracts (*p* < 0.05).

As presented in Figure 3, flavonoid levels varied significantly according to biomass and extraction strategy (*p* < 0.05). For AP (Figure 3A), APE 1:10 reached the highest TFC (860.93 mg QE/100 g), without statistical difference from APM 1:10 (*p* > 0.05), whereas APA 1:20 exhibited the lowest concentration (391.38 mg QE/100 g) (*p* < 0.05).

Among AS extracts (Figure 3B), ASM 1:10 achieved the maximum flavonoid content (1112.31 mg QE/100 g), followed by ASE 1:10 (954.14 mg QE/100 g), both significantly higher than the remaining treatments (*p* < 0.05). Conversely, ASA 1:20 presented the minimum value (227.60 mg QE/100 g) (*p* < 0.05).

For MP (Figure 3C), flavonoid concentrations ranged from 215.92 to 817.83 mg QE/100 g (*p* < 0.05). The highest values were obtained with alcoholic solvents at 1:10, whereas MPA extracts consistently showed the lowest levels (*p* < 0.05). For PP (Figure 3D), PPE 1:10 displayed the highest TFC (403.52 mg QE/100 g), while PPA 1:20 recorded the lowest value (156.48 mg QE/100 g) (*p* < 0.05). The proportional relationship between TFC and TPC observed for AS and AP suggests that flavonoids represent a substantial fraction of total phenolics in these residues. In contrast, the comparatively lower TFC‐to‐TPC ratio verified for MP and PP indicates a more heterogeneous phenolic composition, likely involving non‐flavonoid subclasses.

**FIGURE 4:**
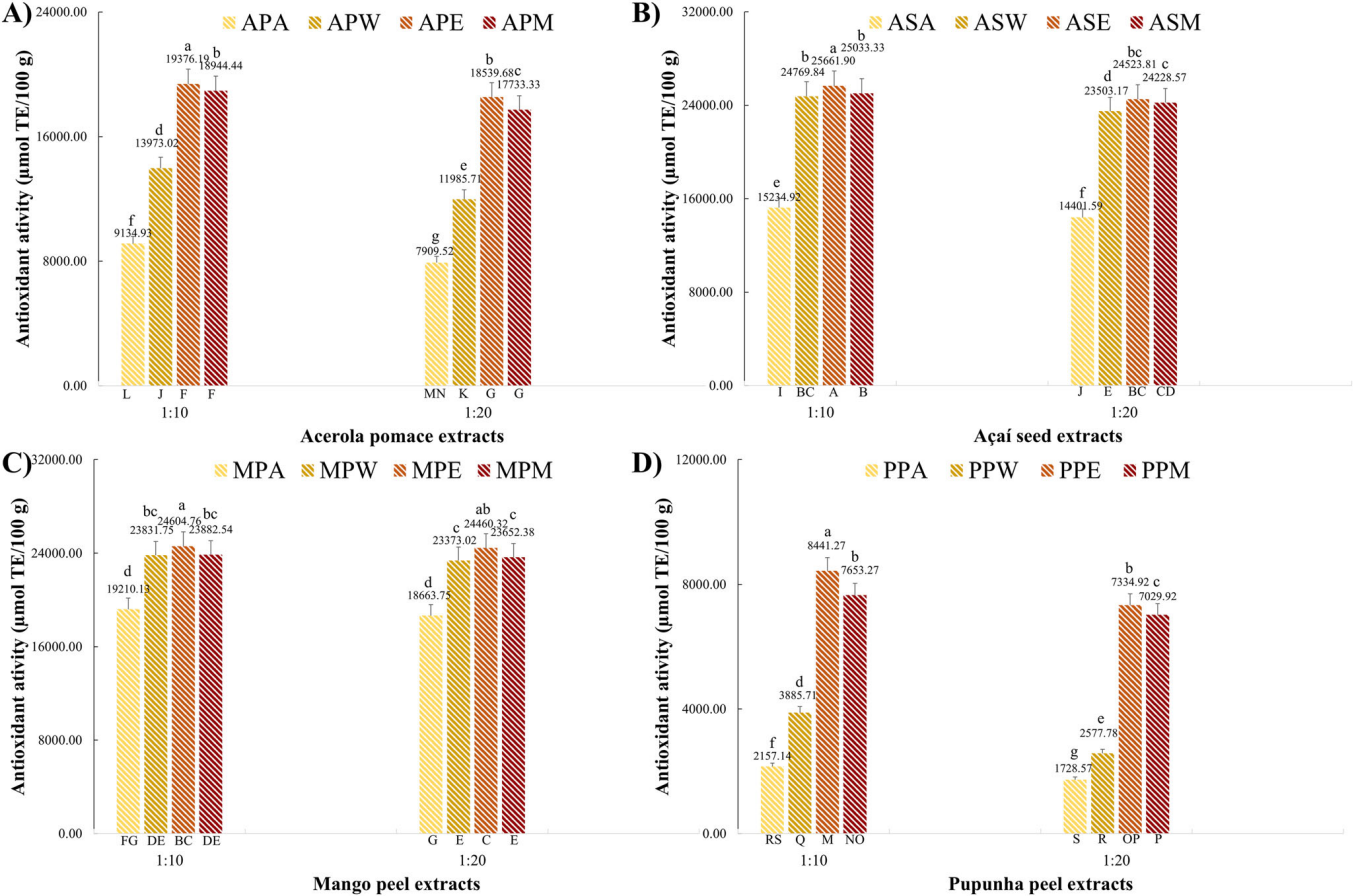
Antioxidant activity (AA) of extracts obtained from Amazonian agro‐industrial residues: acerola pomace (A), açaí seed (B), mango peel (C), and pupunha peel (D). Experiments were presented as mean ± standard deviation. Different lowercase letters (a, b) indicate significant differences (*p* < 0.05) among the means of extracts obtained from the same residue (AP, AS, MP, and PP) by Tukey's test. Different uppercase letters (A, B) indicate significant differences (*p* < 0.05) among the means of the evaluated extracts by Tukey's test. AA, antioxidant activity; APA, acerola pomace acid extract; APE, acerola pomace ethanolic extract; APM, acerola pomace methanolic extract; APW, acerola pomace water extract; ASA, açaí seed acid extract; ASE, açaí seed ethanolic extract; ASM, açaí seed methanolic extract; ASW, açaí seed water extract; MPA, mango peel acid extract; MPE, mango peel ethanolic extract; MPM, mango peel methanolic extract; MPW, mango peel water extract; PPA, pupunha peel acid extract; PPE, pupunha peel ethanolic extract; PPM, pupunha peel methanolic extract; PPW, pupunha peel water extract; TE, Trolox equivalent.

As evidenced in Figure 4, AA varied significantly among residues and extraction conditions (*p* < 0.05), following a distribution similar to that observed for TPC and TFC in AP, AS, and PP, whereas MP extracts did not exhibit the same proportional behavior. Overall, AS extracts displayed the highest DPPH radical‐scavenging capacity, followed by MP, AP, and PP in decreasing order (*p* < 0.05).

For AP (Figure 4A), APE 1:10 showed the highest AA (19,376.19 µmol TE/100 g), whereas APA 1:20 presented the lowest value (7,909.52 µmol TE/100 g), differing from the remaining treatments (*p* < 0.05). For AS (Figure 4B), ASE 1:10 achieved the maximum AA (25,661.90 µmol TE/100 g), significantly higher than ASM 1:10 (*p* < 0.05). In contrast, ASA 1:20 recorded the lowest value (14,401.59 µmol TE/100 g), differing from the other AS extracts (*p* < 0.05).

For MP (Figure 4C), AA ranged from 18,663.75 to 24,604.76 µmol TE/100 g (*p* < 0.05). MPE 1:10 exhibited the highest value, with no difference compared to MPE 1:20 (*p* > 0.05). Notably, MPW extracts displayed antioxidant activity comparable to organic systems, supporting the feasibility of hydrothermal extraction for this matrix, and MPA 1:20 showed the lowest activity (*p* < 0.05). In general, differences in antioxidant performance among MP extracts did not strictly follow TPC and TFC values, suggesting that additional non‐phenolic phytoconstituents in this matrix, such as carotenoids and antioxidant vitamins, may contribute to radical‐scavenging capacity (Xu et al. 2017). For PP (Figure 4.D), PPE 1:10 displayed the highest AA (8441.27 µmol TE/100 g), while PPA 1:20 presented the lowest value (1728.57 µmol TE/100 g), differing from the other PP extracts (*p* < 0.05).

Reported values for TPC, TFC, and AA in AP extracts vary considerably in the literature, reflecting differences in extraction techniques, solvent systems, and expression basis. Compared with the present study, Rezende et al. (2017) reported lower TPC (1279.70 mg GAE/100 g d.b.), TFC (582.86 mg QE/100 g d.b.), and AA (18,028 µmol TE/100 g d.b.) for acidified ethanolic extracts of AP obtained by ultrasound. Similarly, Carvalho Gualberto et al. (2021) reported a lower TPC value (444.05 mg GAE/100 g of dry extract) for acetone extracts obtained by ultrasound. Moreover, da Silva Donadone et al. (2025) reported a higher TPC value (3.36 g GAE/100 g of dry extract) and a lower AA (4970 µmol TE/100 g of dry extract) for ultrasound‐assisted ethanolic extraction. In contrast, Alves Borges et al. (2021) obtained a higher TPC value (10680 mg GAE/100 g of dry extract) using gas‐expanded liquid extraction.

Considering studies that evaluated AS extracts, Buratto et al. (2021) reported lower TPC and TFC values (1770 mg GAE/100 g d.b. and 242 mg QE/100 g d.b., respectively) for ultrasound‐assisted ethanolic extracts, compared to the present work. In contrast, Melo et al. (2021) reported higher TPC value (6458 mg GAE/100 g) and DPPH‐based AA (62,281 µmol TE/100 g) for an optimized ethanolic extract obtained from the same raw material, which may be associated with the freeze‐drying pretreatment applied prior to extraction.

No studies were identified reporting TPC, TFC, or AA values for MP extracts from the ‘Espada’ cultivar, which limits comparisons with our findings. For other cultivars, Guandalini et al. (2019) reported a lower TPC value (1382 mg GAE/100 g d.b.) for MP methanolic extracts of Tommy Atkins. In contrast, Marcillo‐Parra et al. (2021) reported higher TPC values ranging from 2930 to 6624 mg GAE/100 g d.b. for acetonic extracts of MP from the Tommy Atkins, Haden, and Kent cultivars, while the corresponding TFC values were lower in comparison with this study, varying between 502 and 795 mg QE/100 g. Furthermore, Ramirez‐Brewer et al. (2024) obtained higher TPC values (12,130 and 22,486 mg GAE/100 g) for optimized freeze‐dried ethanolic MP extracts produced by microwave‐assisted extraction of Tommy Atkins and Sugar cultivars, respectively. For PP extracts, studies investigating TPC, TFC, and AA contents in the raw material remain scarce, in contrast with a great number of studies focused on carotenoid composition, highlighting the importance of characterizing this residue. Nevertheless, Martínez‐Girón et al. (2024) reported lower TPC values (83.17 ± 1.76 mg GAE/100 g d.b.) for the ethanolic extract of PP.

**FIGURE 5:**
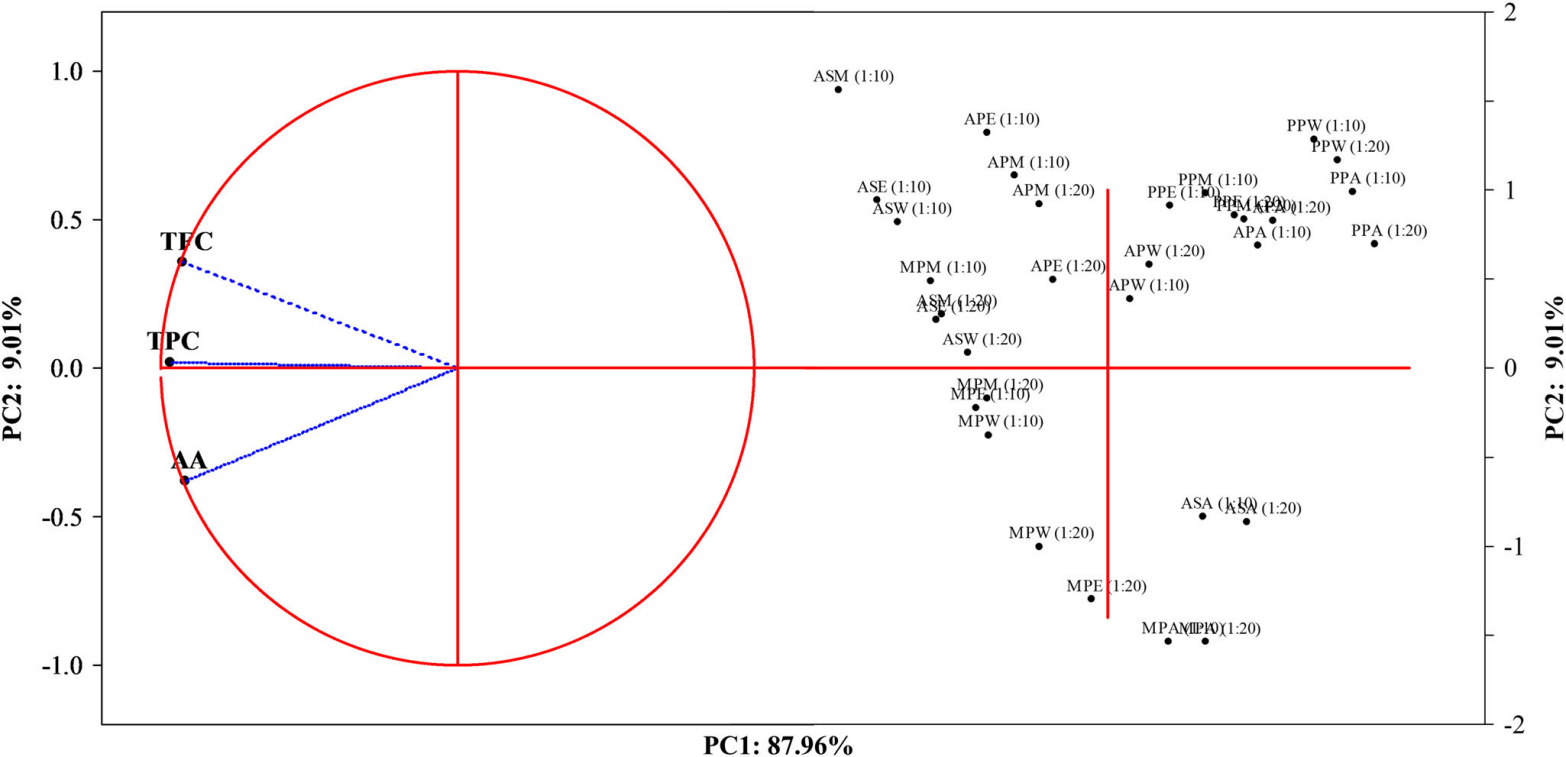
Principal component analysis (PCA) of extracts from Amazonian agro‐industrial residues: acerola pomace (AP), açaí seed (AS), mango peel (MP), and pupunha peel (PP). The figure combines the PCA score plot (sample distribution) and loading plot (variable contribution). APA, acerola pomace acid extract; APE, acerola pomace ethanolic extract; APM, acerola pomace methanolic extract; APW, acerola pomace water extract; ASA, açaí seed acid extract; ASE, açaí seed ethanolic extract; ASM, açaí seed methanolic extract; ASW, açaí seed water extract; MPA, mango peel acid extract; MPE, mango peel ethanolic extract; MPM, mango peel methanolic extract; MPW, mango peel water extract; PPA, pupunha peel acid extract; PPE, pupunha peel ethanolic extract; PPM, pupunha peel methanolic extract; PPW, pupunha peel water extract.

Figure 5 shows the principal component analysis (PCA) with the integration of TPC, TFC, and AA results for AP, AS, MP, and PP extracts. The two components (PC1 and PC2) explain the correlations of TPC, TFC, and AA with a variance of 96.97%. TPC and TFC are grouped in the same quadrant, and AA in the adjacent quadrant, indicating a positive association between the determinations, which tend to show >TPC > TFC > AA. Furthermore, the extracts that are organized on the opposite side show lower results for the bioactive compounds analyzed.

The samples (MP) with negative PC1 and PC2 are closer to AA, suggesting that in some plant matrices, antioxidant activity may be influenced by the broader composition of biomolecules, not just the group of total phenolic compounds. The lower TPC and TF generally observed after acid treatment, compared with alcoholic extraction, further suggest that the treatment severity may have favored degradation or transformation of some bioactive constituents rather than their efficient recovery (Shi et al. 2022).

In general, PCA shows that extractions with methanol and 70% ethanol (especially at a mass/solvent ratio of 1:10) presented higher values of TPC, TFC, and AA, while increasing the solvent volume associated with aqueous and acidic extractions tends to shift the extracts to PCA regions associated with lower values of antioxidant phenolics, possibly justified by the dilution effect and/or the presence of other non‐phenolic bioactive.

However, because only two mass/solvent ratios were evaluated, future studies should investigate intermediate ratios to more accurately determine the optimal extraction conditions for each biomass. In addition, optimization of temperature, time, and acid concentration may improve the extraction efficiency of these treatments.

### 3.2 Profile of Major Phenolic Compounds From ASE by HPLC‐DAD

The qualitative analysis of the phenolic compound profile of ASE 1:10 was recorded by HPLC‐DAD at 280 nm and is shown in Table 1, based on comparison with a commercial standard. Catechin (Peak 1) was the major antioxidant phenolic compound, as determined by the high contents of TPC and AA observed for ASE in Figures 2B and 4B, respectively.

**TABLE 1:** Phenolic compound profile, identification and quantification by HPLC‐DAD of catechin in the açaí seed extract.

Phenolic compounds profile (Chromatogram) 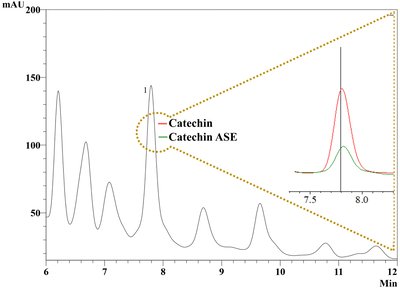	ASE (1:10)	
	Rt (min)	Catechin (mg/100 g)
	7.83 ± 0.05	29.86 ± 0.64

*Note*: Catechin content is expressed as mean ± standard deviation in dry extract.

Abbreviations: ASE (1:10), açai seed ethanolic extract with mass/solvent ratio of 1:10 (w/v); Rt, retention time.

According to the literature, catechin has been reported as one of the predominant phenolic constituents in *E. oleracea* Mart. seed extracts (Martins et al. 2020; Martins et al. 2022). Barros et al. (2015) characterized the phenolic fraction of aqueous açaí seed extract as consisting predominantly of catechin derivatives, with a total quantification of 19.1 ± 0.1 mg/g, whereas Conrado et al. (2025) quantified catechin (1.62  ± 0.09 mg/g) in hydroethanolic extract. However, differences in catechin concentration among studies may reflect variations in sample pretreatment, solvent composition, extraction conditions, analytical methods, and basis used to express the results.

### 3.3 In Vitro Antibacterial Activity Assessment

According to the results presented in Table 2, among all extracts evaluated, only the ethanolic (ASE) and methanolic (ASM) extracts obtained from açaí seeds exhibited antibacterial activity within the tested concentration range, regardless of the mass/solvent ratio (1:10 or 1:20; w/v). Sensitivity was observed for three of the five bacterial strains tested, namely *S. aureus*, *P. mirabilis*, and *P. aeruginosa*. The antibacterial activity observed highlights the application of these extracts in food safety against recognized foodborne pathogens (Wang et al. 2026; Zheng et al. 2026).

**TABLE 2:** Minimum inhibitory concentration (MIC) and minimum bactericidal concentration (MBC) of açaí seed (AS) extracts against pathogenic bacteria.

Sample	Mass/solvent ratio	*Proteus mirabilis*		*Pseudomonas aeruginosa*		*Staphylococcus aureus*	
		MIC	MBC	MIC	MBC	MIC	MBC
ASE	01:10	4 mg/mL	>8 mg/mL	4 mg/mL	>8 mg/mL	1 mg/mL	2 mg/mL
	01:20	4 mg/mL	>8 mg/mL	4 mg/mL	>8 mg/mL	1 mg/mL	2 mg/mL
ASM	01:10	4 mg/mL	>8 mg/mL	4 mg/mL	>8 mg/mL	1 mg/mL	2 mg/mL
	01:20	4 mg/mL	>8 mg/mL	4 mg/mL	>8 mg/mL	1 mg/mL	2 mg/mL

Abbreviations: ASM, açai seed methanolic extract; ASE, açai seed ethanolic extract; MBC, minimum bactericidal concentration; MIC, minimum inhibitory concentration.

For *S. aureus*, both AS extracts showed pronounced antibacterial activity, with MICs of 1 mg/mL and MBCs of 2 mg/mL, indicating a bactericidal effect. In contrast, for the Gram‐negative strains *P. mirabilis* and *P. aeruginosa*, higher concentrations were required to inhibit bacterial growth (MIC = 4 mg/mL), and no bactericidal activity was observed within the tested range (MBC > 8 mg/mL), characterizing a bacteriostatic effect.

These results highlight the greater susceptibility of *S. aureus* to AS extracts, a Gram‐positive bacterium widely recognized for its pathogenicity and strong biofilm‐forming capacity (Howden et al. 2023). The reduced sensitivity observed for Gram‐negative bacteria may be attributed to the structural complexity of their outer membrane, which is rich in lipopolysaccharides and acts as a selective permeability barrier, limiting the penetration and interaction of bioactive compounds with intracellular targets (Leanse et al. 2023).

When compared with previously reported studies, the antibacterial activity observed in the present work shows both similarities and distinctions. Sprenger et al. (2016) reported antibacterial effects of a lyophilized hydroalcoholic extract of *E. oleracea* seeds against *S. aureus* and *P. aeruginosa* using the disk diffusion method, with MIC values of 0.32 and 2.56 mg/mL, respectively. Martins et al. (2020) reported comparable MIC and MBC values for methanolic extracts of açaí seeds against *S. aureus*, using the agar well diffusion method and macrodilution, while no inhibitory activity was detected against Gram‐negative strains, corroborating the selective antibacterial profile observed herein. Similarly, Dias‐Souza et al. (2018) showed strong antibacterial activity of methanolic extracts obtained from açaí pulp against a hospital‐isolated *S. aureus* strain, reporting MIC and MBC values of 0.007 and 0.062 mg/mL, respectively. The markedly higher potency reported in that study may be related to differences in botanical part, phenolic composition, and bacterial strain susceptibility.

In this context, the antibacterial activity observed for açaí seed extracts can be associated with their high content of phenolic compounds. This assumption is supported by TPC values obtained in the present study (Figure 2), suggesting that phenolic constituents may substantially contribute to the observed antibacterial activity. Evidence from the literature indicates that phenolic compounds can exert antimicrobial effects by disrupting bacterial cell membrane integrity and permeability, interacting with intracellular enzymes, interfering with nucleic acid synthesis, or chelating essential metal ions, ultimately compromising bacterial viability (de Rossi et al. 2025; Zhao et al. 2025). In further investigations, bacterial morphological analyses, such as scanning and transmission electron microscopy, may help elucidate the cellular damage and antibacterial mechanisms of AS extracts (Shivakumar et al. 2026). In addition, future studies should include typical food spoilage bacteria and molds to broaden the applicability of these findings to practical food preservation scenarios.

### 3.4 Cytogenetic Safety of Bioactive Extracts

The results presented in Table 3 indicate that cells treated with ASM at both mass/solvent ratios (1:10 and 1:20) exhibited a significantly higher frequency of chromosomal aberrations (99.07% and 97.15%, respectively) compared with the other groups.

**TABLE 3:** Mitotic index, chromosomal aberrations, and cellular alterations in *Allium cepa* meristematic cells exposed to açaí seed (AS) extracts.

				Cellular alterations (*n*) 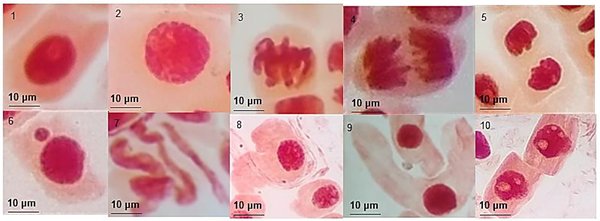				
Sample	Mass/solvent ratio	MI (%)	CA (%)	Micronuclei	Extensive cells	Pycnotic nuclei	Other morphological changes	Cytoplasm destruction
ASE	01:10	35.85 ± 0.10^b^	64.15 ± 0.10^d^	14	177	542	356	727
	01:20	60.73 ± 0.10^a^	39.27 ± 0.10^f^	5	46	297	173	505
ASM	01:10	0.93 ± 0.28^d^	99.07 ± 0.28^a^	106	486	1547	424	2263
	01:20	2.85 ± 0.25^d^	97.15 ± 0.25^b^	23	79	4319	397	1233
PC		8.15 ± 0.48^c^	91.85 ± 0.48^c^	87	297	730	221	928
NC		58.80 ± 2.26^a^	41.30 ± 2.30^e^	5	67	159	34	259

*Note*: Experiments were performed in triplicate, and data are expressed as mean ± standard deviation (SD). Different lowercase letters in the same column indicate statistically significant differences (*p* < 0.05).

Representative images of normal mitotic division in the negative control (NC): (1) interphase, (2) prophase, (3) metaphase, (4) anaphase, and (5) telophase. Cellular alterations observed after 24 h of exposure to ethanolic and methanolic açaí seed extracts at concentrations equivalent to the MBC: (6) micronuclei formation in ASM 1:10; (7) morphological alteration in the positive control (PC); (8) cytoplasmic destruction in ASM 1:20; (9) extensive cytoplasm in ASE 1:10; and (10) pycnotic nuclei in ASM 1:20.

Abbriviations: ASE, açaí ASM, açaí CA, chromosomal aberrations; MI, mitotic index; NC, negative control; PC, positive control (colchicine). seed ethanolic extract; seed methanolic extract.

In contrast, ASE treatments showed lower values, reaching 64.15% for ASE 1:10 and 39.27% for ASE 1:20. Chromosomal aberrations are widely recognized as indicators of genotoxic potential and include structural or numerical chromosomal alterations resulting from DNA damage or inhibition of DNA synthesis (Kumar et al. 2020).

The mitotic index (MI), which reflects the proportion of cells undergoing division and serves as a cytotoxicity parameter, was significantly reduced in cells exposed to ASM 1:10 and 1:20 (0.93% and 2.85%, respectively) when compared with ASE 1:10 and 1:20, which showed higher MI values (35.85% and 60.73%, respectively). When compared with the negative control, ASM treatments clearly exerted a mitodepressive effect on meristematic cells, a phenomenon commonly associated with G1‐phase arrest, inhibition of DNA synthesis, or prolongation of the S phase, ultimately delaying cell division (Sabeen et al. 2020). In addition, the lower MI and higher CA values observed in the ASM‐treated groups compared with the positive control (MI of 8.15% and CA frequency of 91.85%) reinforce the higher cytotoxic and genotoxic potential of the ASM.

In contrast, treatment with ASE 1:20 did not result in significant differences (*p* > 0.05) compared with the negative control (MI of 58.80% and CA of 41.30%), which contained 1% DMSO, a concentration previously reported as non‐cytotoxic and non‐genotoxic (Sivaram et al. 2018). Therefore, at the lowest concentration required to achieve bactericidal activity (2 mg/mL), ASE 1:20 demonstrated a more favorable cytogenetic safety profile. Additionally, while the mass/solvent ratio did not influence MI and CA values for ASM, differences were observed for ethanolic extracts (*p* < 0.05), indicating that extraction yield and biomass loading affected the biological response.

Although phenolic compounds have been associated with cytotoxic and genotoxic effects due to their allelopathic role in plant defense mechanisms (Hoyberghs et al. 2021), this relationship was not proportional in the present study.

Despite similar TPC values (Figure 2), ASM and ASE exhibited different mitotic indices (*p* < 0.05), suggesting that factors beyond TPC contributed to the observed toxicity. In this context, the higher cytogenetic impact of ASM may be related to the polarity and solubility of phytochemicals preferentially extracted by methanol. Previous studies have reported that methanolic extracts are more susceptible to inducing DNA damage due to their efficiency in extracting highly polar compounds; however, this effect is strongly dependent on extraction conditions, raw material characteristics, and exposure time (Duarte et al. 2024).

The main cellular alterations observed in treated cells included cytoplasmic destruction, morphological alterations, pycnotic nuclei, cells with extensive cytoplasm, and micronuclei formation (Table 3). Notably, treatments with ASM resulted in a higher frequency of abnormal cells, corroborating the increased chromosomal aberrations and reduced mitotic index observed in this group. In addition, cells treated with ASM 1:10 exhibited a higher incidence of micronuclei, a well‐established biomarker of mutagenicity that reflects irreversible DNA damage (Al‐Naqeb et al. 2024). Because cytogenetic safety was evaluated at a single concentration (2 mg/mL), selected based on the bactericidal activity against *S. aureus*, these findings should be considered preliminary. Nevertheless, they provide a starting point for investigating the dose‐dependent toxicological profiles of AS extracts and their use as antimicrobial agents.

Overall, these results indicate that ASE is the most promising extract for food preservation applications among the Amazonian fruit residues evaluated, given its high antioxidant activity, antibacterial effect, and favorable cytogenetic safety profile at the bactericidal concentration. Phenolic‐rich extracts have been increasingly investigated as natural alternatives to synthetic additives, owing to their ability to delay lipid oxidation and inhibit microbial growth, thereby improving oxidative stability and extending shelf life in food systems, as well as their incorporation into edible coatings, active packaging materials, and biodegradable films (Andrade et al. 2025; Gutiérrez‐del‐Río et al. 2021; Oulahal and Degraeve 2022). In this context, the biological properties demonstrated in the present study support the future evaluation of ASE in such application scenarios. Nevertheless, further studies in real food matrices are still required to assess its technological performance, storage stability, and interactions with food components prior to practical implementation.

## 4 Conclusions

Comparative evaluation of Amazonian fruit residues revealed that açaí seed (AS) exhibited the highest bioactive potential among the investigated biomasses, followed by mango peel (MP), acerola pomace (AP), and pupunha peel (PP). In general, ethanolic and methanolic extractions at a 1:10 (w/v) mass/solvent ratio maximized phenolic compounds, flavonoids, and antioxidant activity, while hydrothermal extraction showed promising performance for MP, highlighting the potential of water as a sustainable extraction solvent.

Only ethanolic and methanolic AS extracts exhibited antibacterial activity, showing bactericidal effects against *S. aureus* and bacteriostatic activity against Gram‐negative strains. Cytogenetic analyses demonstrated that methanolic AS extracts induced pronounced genotoxic and mitodepressive effects, whereas the ethanolic extract, particularly ASE 1:20, showed a more favorable safety profile at the bactericidal concentration. Future studies should integrate complementary antioxidant methods, such as ABTS and FRAP, and evaluate the thermal and storage stability of the extracts to support their development as natural food additives.

Overall, the findings demonstrate that extracts from Amazonian fruit residues, especially açaí seeds, obtained with 70% ethanol, are promising sources of antioxidant and antibacterial compounds, reinforcing their valorization as high‐value‐added bioproducts within sustainable waste management and bioeconomy strategies.

## Author Contributions

**Lucas Figueiredo da Silva**: conceptualization, writing – original draft, writing – review and editing, visualization, software, methodology, formal analysis. **Yasmin Lobato Salgado**: conceptualization, writing – original draft, writing – review and editing, methodology, visualization, formal analysis, software. **Yanka Braga Palheta**: conceptualization, methodology, visualization, writing – review and editing, writing – original draft, software, formal analysis. **Marta Chagas Monteiro**: validation, writing – review and editing, project administration, supervision. **Ingryd Rodrigues Martins**: validation, supervision, project administration, writing – review and editing. **Johnatt Allan Rocha de Oliveira**: validation, project administration, writing – review and editing, supervision.

## Conflicts of Interest

The authors declare no conflicts of interest.

## Data Availability

The data that support the findings of this study are not publicly available.
